# Psychiatric disorders and comorbidity in women with Turner Syndrome: a retrospective national cohort study

**DOI:** 10.1038/s41398-024-02937-5

**Published:** 2024-09-04

**Authors:** Sofia Thunström, Ulla Wide, Kerstin Landin-Wilhelmsen, Kerstin Berntorp, Inger Bryman, Emily Krantz, Jeanette Wahlberg, Bertil Ekman, Magnus Isakson, Anders Karlsson, Ingrid Bergström, Sabine Naessén

**Affiliations:** 1 The Department of Internal Medicine and Clinical Nutrition, Institute of Medicine, The Sahlgrenska Academy, University of Gothenburg, Gothenburg, Sweden; 2 Department of Clinical Genetics and Genomics, Sahlgrenska University Hospital, Gothenburg, Sweden; 3 Institute of Odontology, Sahlgrenska Academy, University of Gothenburg, Gothenburg, Sweden; 4 The Section of Endocrinology, Sahlgrenska University Hospital, Gothenburg, Sweden; 5 Genetics and Diabetes Research Unit, Department of Clinical Sciences Malmö, Lund University, Malmö, Sweden; 6 Department of Obstetrics and Gynecology, Institute of Clinical Sciences, Sahlgrenska Academy at the University of Gothenburg, Gothenburg, Sweden; 7 Department of Respiratory Medicine, Sahlgrenska University Hospital, Gothenburg, Sweden; 8 Department of Medicine, Örebro University Hospital, Örebro, Sweden; 9 School of Medical Sciences, Faculty of Medicine, and Health, Örebro University, Örebro, Sweden; 10 Department of Endocrinology, Linköping University Hospital, Linköping, Sweden; 11 Department of Medical Sciences, Uppsala University, Uppsala University Hospital, Uppsala, Sweden; 12 Department of Clinical Science, Intervention and Technology, Karolinska Institutet, Solna, Sweden; 13 Department of Women’s and Children’s Health, Karolinska Institutet, Stockholm, Sweden; 14 Academic Primary Health Care Centre, Region Stockholm, Stockholm, Sweden

**Keywords:** Depression, Bipolar disorder

## Abstract

Turner syndrome (TS) is a genetic condition characterized by partial or complete monosomy X. A reduced life expectancy has been shown in TS, depending on an increased risk of aortic dissection, and ischemic heart disease. Studies covering the occurrence of psychiatric conditions are sparse within TS. Several case reports describe concomitant TS and neuropsychiatric abnormalities that may represent a pathogenetic link to genetics, as well as feature correlates of TS. The aim of this study was to determine the presence, and the frequency of psychiatric diagnosis in women with TS in a Swedish cohort followed during 25 years’ time. Statistics from the entire female population in Sweden of corresponding age was used as reference. Data were retrieved from clinical examinations and validated from the National Board of Health and Welfare registries for women with TS (*n* = 487), aged 16 to 84 years, with respect to mental health disorders. The most common diagnoses in TS were mood and anxiety disorders. There was no increase in psychiatric diagnosis within the group with time, nor correlation to specific karyotype or somatic comorbidity as congenital heart disease and hypothyroidism, hormonal treatment, or childbirth. In addition, the frequency of psychiatric diagnosis in TS was lower than in the population-based data. Further investigations are needed in the view of the fact that women with Turner syndrome should not be burdened with more severe diagnoses.

## Introduction

Turner syndrome (TS) is the most common chromosomal aneuploidy, affecting 1:2.500 live female births. It is a result of complete or partial absence of one of the sex chromosomes, X, and frequently accompanied by a mosaic cell-line. Of girls and women diagnosed with TS, about half are monosomic for the X chromosome (karyotype 45,X). The rest consists of the mosaic karyotypes which describes multiple cell lines co-occurring within the same individual, (45,X/46,XX; 45,X/46,XY; 45,X/47,XXX, or an X chromosome with an aberrant second sex chromosome i.e., ring chromosome, isochromosome or partial deletion) [1, 2].

TS is sometimes classified as a disorder of sex development (DSD) within a cluster of diagnoses involving atypical gonadal and pubertal development. Despite a wide spectrum of variation within the TS, the existing suggestion is that the absence or mosaicism of one X chromosome often presents with a specific pattern of cognitive strengths and deficits [3–6]. Furthermore, the genetic factors have been pointed out to have an impact on the development of several neuropsychiatric disorders, including anxiety and depression [2, 4, 7–9]. Although TS is not associated with any psychiatric syndrome, the different features specific to TS also seem to place individuals at biologically greater risk for depression than individuals unaffected by TS [1, 9–11]. In addition, several case reports and a study have described associations of TS with schizophrenia, and schizophrenic psychoses [12–17].

Major depressive disorder occurs about twice as often in women in the general population than it does in men and affects one in six adults in their lifetime [18–20]. As for depression disorder, women are significantly more likely to develop anxiety disorder throughout the lifespan than men [20–22]. A higher lifetime prevalence rate of depression was found in women with TS, 36%-65%, compared with the community rate of 21% [2, 18, 23]. Though the results obtained by Cardoso et al, was during the discontinuation of hormone replacement therapy, and the sudden hormone fluctuations may have affected the mood and thereby the results [2].

Research conducted on TS with mood disorders is scarce, and the few published results of small studies and case reports are heterogeneous. Hence, the prevalence of bipolar disorder in individuals with TS is still unknown [2, 24–26].

The knowledge about the prevalence of mental health issues in women with TS are limited. The aim of this study was to determine the presence and the frequency of psychiatric diagnosis/disorders in women with TS in a Swedish cohort followed during 25 years’ time.

## Study design and participants

### Subjects

Women with diagnosed Turner syndrome (TS), ≥16 years, were recruited from the Turner Centers, by referral from hospitals or through transition from pediatric clinics, other colleagues or via the national patient society in Sweden. All women provided informed consent. Around one third of all had received growth hormone and 80% had ongoing hormone replacement with estrogen in combination with progesterone without supplemental androgens (Table 1). The participants were monitored every 5th year according to the current national and international guidelines for care and treatment of women with TS [1, 27]. The examinations included medical history, parity, ongoing medications, physical examinations by an endocrinologist and a gynecologist, blood pressure, anthropometric data, blood samples, audiometry, bone mineral density measurement and echocardiography.

**Table 1:** Characteristics of women with Turner syndrome (TS) with and without any psychiatric diagnoses.

Diagnoses	TS with psychiatric diagnosis, *n* = 71	TS without psychiatric diagnosis, *n* = 416	P-level	All women with TS, *n* = 487
Age, years	39.6 ± 8.8	39.0 ± 14.8	0.740	38.8 ± 5.6
Age at TS diagnosis, years	0 ± 6.0	0 ± 9.1	1.0	0 ± 9.0
Height, cm	154.2 ± 18.9	153.9 ± 7.2	0.812	153.3 ± 8.4
Body weight, kg	58.4 ± 12.3	59.8 ± 12.8	0.392	59.5 ± 11.0
Spontaneous puberty	8/71 (11.3%)	51/416 (12.3%)	0.135	59/487 (12.1%)
GH, *n* (%)	35/71 (49.3%)	160/416 (38.5%)	0.085	160/487 (32.8%)
MHT, *n* (%)	67/71 (94.4%)	388/416 (93.3%)	0.730	388/487 (79.6%)
Karyotype				
Monosomy, *n* (%)	49/71 (69.0%)	265/416 (63.7%)	0.387	265/487 (54.4%)
Mosaic form, *n* (%)	9/71 (12.6%)	70/416 (16.8%)	0.381	70/487 (14.3%)
Isochromosome, *n* (%)	8/71 (11.2%)	59/416 (14.1%)	0.819	59/487 (12.1%)
Y-chromosome, *n* (%)	5/71 (7.0%)	22/416 (5.2%)	0.551	22/487 (4.5%)
Iso-Y, *n* %	13/71 (18.3%)	81/416 (19.4%)	0.819	81/487 (16.6%)
Cardiovascular ailments, *n* (%)	13/71 (18.3%)	98/416 (23.5%)	0.330	98/487 (20.1%)
Bicuspid aortic valve, *n* (%)	8/71 (11.3%)	64/416 (15.4%)	0.366	64/487 (13.1%)
Coarctatio aortae, *n* (%)	4/71 (5.6%)	29/416 (7.0%)	0.679	29/487 (5.9%)
Hypertension, *n* (%)	13/71 (18.3%)	50/416 (12.0%)	0.144	98/487 (20.1%)
Hypothyrosis, *n* (%)	20/71(28.1%)	150/416 (36.0%)	0.197	150/487 (30.8%)
Diabetes mellitus, *n* (%)	4/71 (5.6%)	16/416 (3.9%)	0.483	16/487 (3.2%)
Alcohol abuse, intoxication, *n* (%)	6/71 (8.4%)	0	< 0.0001	6/487 (1.2%)
Partnership, *n* (%)	32/71 (45.0%)	164/416 (39.4%)	0.370	164/487 (33.7%)
Child/birth, *n* (%)	11/71 (15.5%)	50/416 (12.0%)	0.414	98/487 (20.1%)

*GH* previous growth hormone (GH) treatment, *MHT* ongoing menopausal hormone therapy. Means + /− SD, *n* (%).

### Methods

The diagnosis of TS was cytogenetically verified by chromosome analyses at birth, at short stature, at absent or delayed puberty/secondary amenorrhea or by other phenotypically clinical signs of TS. Medical records were obtained, and personal identification numbers were used to identify psychiatric diagnosis between 1994 and 2021 from the Swedish National In-, and Outpatient Register and the National Board for Health and Welfare, Stockholm, Sweden.

Every inhabitant appearing in this register is unique either as an individual or as a patient. Each subject with a specific diagnosis can be distinguished from this register based on the various diagnostic codes. For example, women with TS who have a psychiatric diagnosis do not appear in the register again among the women with TS without psychiatric conditions.

Table 2 shows the definitions of various psychiatric disorders within the international classification of diseases (ICD), ICD-9 codes 290–319, E950-E959, and ICD-10 codes F10-F99, X60-X84 [28].

**Table 2:** The definitions of various psychiatric disorders within ICD-8,9 codes 290–315, ICD-9 codes 290–319, and ICD-10 codes F10–F99.

Diagnosis	ICD-10 codes, > 2001	Equivalent ICD-8,9 codes, 1987–2001
Any neurodevelopmental disorders/psychiatric disorders	F10-F99	290–319
Mental and behavioral disorders due to psychoactive substance use	F10-F19	291, 303, 304
Schizophrenia and related disorders (including schizophrenia schizoaffective disorder)	F20-F29	295, 297, 298
Mood disorders (including bipolar, single, and recurrent depressive disorder)	F30-39	296, 300E, 311
Anxiety disorders (including dissociative, stress-related and somatoform disorders)	F40-48	300, 300A-300D, 300F-300X, 308–309
Eating disorders	F50	307B, 307 F
Disorders of adult personality and behavior	F60-F62, F69	301
Mental retardation (ID)	F70-F79	317–319
Autism spectrum disorders (ASD)	F84-F84.9	299
Hyperkinetic disorders (ADHD)	F90	314
Suicide attempts	X60-X84	E950-E959

Diagnostic and Statistical Manual of Mental Disorders (DSM-5) is an international diagnostic manual, although the National Board of Health and Welfare in Sweden advocates the ICD codes, currently ICD-10. ICD diagnostic system was published by the World Health Organization (WHO) in 1990 and will soon be updated to ICD-11 [28]. The 9^th^ edition of ICD-9 was adopted by WHO in 1976, and from 1987 the ICD 9 was in use. Overall, the two different systems, ICD, and DSM-V, tend to conform relatively well, even though the two systems differ somewhat, especially regarding the autism spectrum disorders. Nevertheless, healthcare professionals do use both systems for the diagnostic criteria.

### Statistical analyses

Means and standard deviations were calculated with conventional methods. Tests between groups were performed by Students´ t-test for continuous data, and the Chi^2^ test for dichotomous variables, GraphPad Software. Two-sided *p* values < 0.05 were considered to be statistically significant. All analyses were performed using either SPSS version 24.0 for Windows (IBM, Armonk, NY, USA) or SAS version 9.4 (SAS Institute Inc., Cary, NC, USA).

### Ethical approvals

This study was approved by the Regional Ethical Review Board in Gothenburg Dnr 456–1994, Dnr S572-1999, Dnr 242–2002, Dnr 088–2006, and in Stockholm for the National project Dnr 660–2009, Ö5-2010 and completed for the Registry study Dnr 01127-2021.

## Results

### Women with TS with and without psychiatric diagnosis

A total of 487 women with TS were included in the study. There were 71 with (14.6%), and 416 (85.4%) without psychiatric disease (Table 1). There were no differences between the groups of women with TS with and without psychiatric diagnosis concerning age, age of diagnosis, karyotype, spontaneous puberty, presence of cardiovascular diseases, endocrine conditions, partnership or whether the individuals had given childbirth, respectively (Table 1).

However, in the group of women with TS and psychiatric disease six individuals (8.5%) had alcohol dependence and behavioral disorders due to psychoactive substance use. Thus 1.2% of the entire group with TS had received hospital care more than once due to alcohol intoxication, delirium tremens, and all had comorbid psychiatric disorders, including mood and behavioral disorders. Smokers and/or former smokers in both groups were rare (data not shown).

### Psychiatric diagnosis in TS and in the general population

Table 2 shows the psychiatric diagnoses according to the ICD codes used for this study. Table 3 shows the distribution of psychiatric diagnoses according to the diagnostic codes in women with TS and in relation to women of similar age in the general population. The most common psychiatric diagnoses in both TS and in the general population were mood disorders followed by the anxiety disorders, with 9.9% and 7.4% for the entire group with TS, respectively, versus 36% and 11–41% in the female population, respectively, *p* < 0.00001 and *p* = 0.11-*p* < 0.00001, respectively.

**Table 3:** Diagnostic codes according to ICD-10, prevalence of psychiatric diagnoses in the study group of women with Turner syndrome (TS) (n = 487) and prevalence in the general population of women with corresponding age.

Diagnosis	ICD-10 codes	Prevalence in women with TS	Prevalence of psychiatric diagnoses in female population in Sweden^a^	*P*-level TS vs population
Mental and behavioral disorders due to psychoactive substance use	F10.1 F10.4	7/487 (1.4%)	20–30%	*P* < 0.00001
Schizophrenia and related disorders (including schizophrenia schizoaffective disorder)	F20.0 F25.2 F29.0	3/487 (0.6%)	0.4%	*P* < 0.00001
Mood disorders (including bipolar, single, and recurrent depressive disorder)	F30.2	47/487 (9.9%)	36%	*P* < 0.00001
Anxiety disorders (including dissociative, stress-related and somatoform disorders)	F40.0 F40.1	36/487 (7.4%)	11% severe and 41% moderate to mild	*P* = 0.11–0.0001
Eating disorders (anorexia nervosa)	F50.0	2/487 (0.4%)	2.4%	*P* = 0.004
Disorders of adult personality and behavior	F60.3 F60.2	8/487 (1.6%)	5%	*P* = 0.0006
Mental retardation (ID)	F70.9 F71.1	3/487 (0.6%)	0.5–1%	*P* = 0.717–0.395
Autism spectrum disorders (ASD)	F84.5 F84.8	3/487 (0.6%)	1%	*P* = 0.395
Hyperkinetic disorders (ADHD)	F90.0B F90.C	5/487 (1.0%)	7–10%	*P* < 0.00001
Suicide attempts	X60.99	2/487 (0.4%)	276/100000/year 0.3%	*P* = 0.572
Suicide	X60.99	1/487 (0.2%)	331/100000/year 0.3%^b^	*P* = 0.630

^a^Foot note: Report from the Swedish public health authority on psychiatric diagnoses, 2021.

^b^Data applies to individuals aged 16–84 years of age.

P-level depicts comparison between women with TS and women in the general population.

Of the 47 cases with mood disorders, 30 had a comorbid psychiatric diagnosis (42.3%). Of the women with TS and psychiatric disease, 65% were also found to have comorbid psychiatric disorders, while 35% had single psychiatric diagnosis.

Six individuals with TS had alcohol abuse/addiction, and five of them attention deficit hyperactivity disorder (ADHD) diagnoses which comprising 1.6% and 1.0% of the whole cohort of TS, respectively (Table 3).

Three cases of schizophrenia were identified, comprising 4.2% of the women with TS and psychiatric disease, and 0.6% of the total cohort of TS which was the only psychiatric diagnosis that was more prevalent in TS than in women in the general population, 0.4%, *p* < 0.00001. Otherwise, the prevalence of psychiatric diagnoses in TS were lower, or similar, to women in the general population in Sweden (Table 3).

In total there were five cases of psychosis in this material of TS, and two cases were connected to bipolar disorder, one with developmental disorder, and the other one had generalized anxiety disorder. Of all five, only one had a somatic illness (hypothyroidism).

There were two cases of anorexia nervosa, comprising 2.8% of the group with TS and psychiatric diseases. Anorexia nervosa constituted 0.4% of the entire cohort of TS, which was lower than in the general population, *p* = 0.004 (Table 3).

Within the group of TS with psychiatric disorder, there were six cases with ADHD, four of them with comorbid psychiatric conditions, and three cases of autism spectrum disorders. Two of them had comorbid diagnosis of schizophrenia, both with karyotype 45,X. One woman with mental retardation and karyotype mosaicism had diabetes mellitus type1. During the observation period, one woman with TS without any psychiatric diagnosis, committed suicide after an acute life crisis, not significantly different from the women in the general population (Table 3).

## Discussion

The main findings of this study were that women with TS in general had lower frequency of psychiatric diagnosis than those obtained from women of similar age in the general population-based register, and that there were no associations between psychiatric diagnoses, other comorbidity, or karyotype in TS, respectively.

TS has been associated with a range of psychiatric conditions such as anxiety, depression, and schizophrenia [2, 7, 29]. The most common psychiatric diagnoses found in the present study were *mood and anxiety disorders*, followed by *disorders of adult personality and behavior*. The frequency of depression diagnosis during more than two decades was less than 10%. Apart from the fact that depression is more common in women in general, women are also diagnosed with more psychiatric comorbidities than men [18, 30]. The life-time risk of developing depression for women at some point in life is 36% in Sweden [31]. Although higher lifetime prevalence rate of depression was found for the entire group of women with TS, the frequency of depression diagnosis was 9.9%, which is far below the community rate (Table 3). Further, both anxiety, and depression can occur as part of other psychiatric disease processes [32]. Anxiety disorders are the most common diagnosis of mental disorders, affecting more often women with greater illness burden [21–23]. The conditions are often long-lasting, and the risk of relapse is high [33]. The reported prevalence of anxiety in Swedish female population varies between 11% (very severe anxiety) to 41% (moderate to mild form) [31]. The prevalence of anxiety disorders in the study group of TS was considerably lower than the severe forms in the general population. It is rational, and expected, that the comorbidity of a second psychiatric condition along with depression would be more common than co-occurrence of depression with TS due to the overlapping biochemical processes and predisposing life events related to psychiatric diseases. Thus, sex differences in psychiatric disorders may be a result of a complex interplay between genetic, hormonal, immunological, and psychosocial factors [34–36]. Mortality rates among people treated for depression and anxiety disorders is 70% higher compared to the rest of the population, in Sweden [31]. This was, however, not seen for women with TS in the present assessment. During the observation period, one woman with TS without any psychiatric diagnosis committed suicide after an acute life crisis.

The different features specific to TS place individuals at biologically greater risk for depression than individuals unaffected by TS [37, 38]. In addition, depression has also been observed in populations with shared characteristics, such as chronic medical conditions [39–41]. Autoimmune diseases seem to play a causative role in several neuropsychiatric disorders [1, 42–44], and generally more prevalent in individuals with chromosome aberrations than in the general population [42, 45] with variable prevalence in TS [46, 47]. However, in the present study no significant differences were found between women with TS and those with or without psychiatric diagnoses with respect to autoimmunity, aortic dissection, ischemic heart diseases, and chronic medical conditions. Neither were there any differences regarding psychiatric disease in TS with or without spontaneous puberty, respectively. It has been shown that neuroactive steroids seem to play a role in mood disorders [38, 40, 48, 49]. The gonads and adrenal cortex are the primary sources of testosterone in both sexes. Individuals with TS have a relative testosterone deficiency because of absence of ovaries [50, 51]. Low testosterone levels in women with TS may be a risk factor for psychiatric illness, as testosterone supplementation has been shown to have anxiolytic and antidepressant effects in women [11]. A randomized, double-blind, placebo-controlled crossover study has shown improved body composition, neurocognition, and quality of life in TS with androgen substitution [52].

A recent study by Rosenberg et al., highlights the problem with fatigue in women with TS, without apparent association with somatic health disorders. The authors hypothesized that stress and neuropsychological processes like deficiencies in executive functioning may play an important role in the etiology of fatigue in women with TS [53]. Difficulties with visual-spatial reasoning, visual-spatial memory, attention, executive functioning, and motor skills seem more common in girls with TS than the general population [8, 38]. An increased risk of ADHD and autism spectrum disorders in girls with TS and register studies has been suggested [6, 54]. The aim of the present study was to evaluate the frequency of psychiatric disorders in adult women with TS only. No systematically recording of fatigue complaint was performed in this national cohort. However, a subgroup has reported similar scores in both physical and psychological subscales for health-related quality of life, except for social isolation, as women in the general population [55]. The regular clinical and thorough examinations for up to 25 years might have contributed to this general well-being in TS.

There are additional potential factors, as mentioned below, that can contribute to an increased risk of mental illness in women with TS. For example, fertility issues are an important factor in emerging depression in women with TS. The ovarian dysgenesis and hypergonadotropic hypogonadal state, results in infertility in the great majority of women with TS [50, 51]. It has been shown that, patients with infertility issues are more likely to suffer from a psychiatric illness than fertile patients [56–58]. A nearly equivalent lifetime rate of a major depressive diagnosis was found in the women with TS as compared to women with ovarian insufficiency (55%), a condition with some shared hormonal and psychosocial factors [59].

To our knowledge, there are no studies on alcohol use in women with TS. Kagan-Krieger et al, found no association between paternal or maternal alcohol consumption and TS [60]. As seen in the present study, problems related to alcohol consumption were also related to the presence of comorbid psychiatric disorders. The individuals with TS who had alcohol addiction had received hospital care more than once due to alcohol intoxication and delirium tremens. Further, publications about TS and eating disorders, especially anorexia nervosa, is extremely rare though there are case reports [61, 62].

In the clinical settings the younger generation of women with TS point out lack of psychological support [63]. Even though there are numerous potential risk factors for depression related to the biological and psychosocial characteristics of TS, higher rate of social skills difficulties than age-matched girls from the general population [6] or increased frequency of depression could not be confirmed in this study. Having TS does not necessarily imply more morbidity in mental illnesses. Reported neuropsychological profile in women with TS, can most likely contribute to challenges in various aspects of daily life including social interplay at work and leisure, and emotional functioning. These difficulties might create anxiety and maybe even lead to anxiety/depression in vulnerable individuals. As somatic health care improves for women with TS, more emphasis should be laid on adolescent years, psychological interventions on issues with self-esteem, body image, and possible difficulties in daily life.

Studies on mental health issues among women with TS are scarce. Many women with TS have normal intelligence, social functioning and has employment, yet there are case reports of psychiatric disorders in this syndrome. Given the frequency of TS, and the existing data, these questions warrant more scientific investigation to find evidence if the chromosomal abnormalities lead more often to psychiatric illness. If so, it is important to identify it to avoid labeling women with a more severe diagnosis and having optimal treatment strategies.

*The strength of the study* is the long-term follow up with clinical examinations of all women with TS during up to 25 years in Sweden. Medical records along with other clinical characteristics of all participants and data from national registries were studied. All listed diagnostic codes are consistent since the reporting to the National Board of Health and Welfare’s health data register is compulsory and regulated by law. Health care is publicly funded with universal access to both primary care and non-primary care in Sweden (66). Another strength is the knowledge of exact karyotypes for all participating women with TS in the study. Women with TS and psychiatric diseases could also be compared with their peers without any psychiatric conditions regarding medical history and anthropometry, (Table 1).

*Limitations* were that there was lack of data on family history of psychiatric diseases in TS, Furthermore, no clinical data were available on the population-based control subjects.

## Conclusion

During follow up of women with TS for more than two decades, no overrepresentation of psychiatric diagnosis was found in these women compared to women in the general population in Sweden. On the contrary, the prevalence of most of the common psychiatric diagnoses was lower in TS than in the population-based data, and there was no association to somatic comorbidity or any karyotype in TS. Further investigations are needed in the view of the fact that women with TS should not be burden with more severe diagnosis.

## Data Availability

The data that support the findings of this study are available on request from the corresponding author, Sabine Naessén.
